# Supplementary material for: Significance of two transmembrane ion gradients for human erythrocyte volume stabilization
Source: PLoS One. 2022 Dec 21;17(12):e0272675. doi: 10.1371/journal.pone.0272675 (PMC9770400; doi:10.1371/journal.pone.0272675)
Supplement: Attachment — Submitted filename: RespToRev.docx [file pone.0272675.s001.docx]

We are grateful to the Reviewers for their valuable comments and suggestions which, to our opinion, caused a significant improvement of the manuscript. Below are our responses to the Reviewer’s comments.

Reviewer #1

The paper by Ataullakhanov et al entitled “Significance of two transmembrane ion gradients for human erythrocyte volume stabilization” is a mathematical modeling study of the role of ionic gradients in stabilization of erythrocyte volume. Maintenance of an optimal surface-to-volume ratio is vital for the physiological function of erythrocytes as it allows them to deform easily inside the capillaries that are several-fold smaller in diameter than the cell if it were a sphere. This manuscript focuses on the benefits of having two (Na+/K+) transmembrane gradients instead of just one (Na+) and demonstrates that larger dynamic range of Na+ concentrations provided by the activity of Na+/K+-ATPase is critical for RBC volume stabilization under several-fold increases in non-specific membrane permeability to ions. The study is very interesting and has applications for other cells, such as neurons where large dynamic range of transmembrane ionic gradients is similarly physiologically vital. I have several suggestions that in my opinion will improve the manuscript and make it more accessible for wider audiences.

Major:

*1. While the model describes transmembrane osmotic gradients, electrical component of ionic gradients is not discussed in the main text. The model does incorporate an algebraic equation for electroneutrality inside the cell and the dependence of passive ion fluxes on membrane potential, but it appears that erythrocyte membrane potential is a constant rather than the variable, which is a significant limitation of this study that the authors should discuss.*

The erythrocyte transmembrane potential is not a constant in the model. It is associated with the distribution of Cl^-^ ions across the cell membrane because the cell membrane permeability for Cl^-^anions in the erythrocyte is two orders of magnitude higher than for cations [1]. In the presence of two transmembrane ion gradients an increase in cell membrane permeability to cations causes a redistribution of Na^+^ and K^+^ ions across the cell membrane while the distribution of Cl^-^ anions and transmembrane potential almost do not change. However, in the case of only one transmembrane ion gradient the transmembrane potential changes dramatically with the change in the cell membrane permeability to cations. We added corresponding explanations and figure illustrating the behavior of transmembrane potential to the manuscript (lines 273-290, Fig. 5).

*2. As the focus of the manuscript is on S/V stabilization, I am curious to see how robust the model is to account for natural cell-to-cell variability. For example, how well is S/V maintained when Na+/K+-ATPase surface density is changed or when intracellular “passive” osmotic pressure is varied?*

Variation of most model parameters do not affect stabilization of erythrocyte cell volume significantly. We added a new paragraph (lines 291-311) and a figure (Fig. 6) that show that the cell volume is stabilized even if Na/K-ATPase or hexokinase activity increase or decrease two times compared with the normal value. However, the erythrocyte cell volume is not stabilized and changes proportional to a content of impermeable molecules in the cell or proportional to osmolarity of the medium that confirms the reputation of the human erythrocyte as an ideal osmometer (lines 312-316, Fig. 7).

Minor:

*1. I don’t see much added value in the 3rd model (Na+/K+-ATPase without Na+-mediated activation). The authors should consider moving these data to supplementary materials or removing them from the manuscript.*

The main role of this model is to demonstrate that the volume stabilization in the case of one transmembrane ion gradient with the regulated transport Na-ATPase is almost as bad as with unregulated Na-ATPase. We added this explanation to the text (lines 200-204) and prefer to keep the 3-d model version in the main manuscript.

*2. Can the authors speculate on the effect of Gardos channels in this model? Shouldn’t the opening of Ca2+-sensitive K+ channels without a change in Na+ conductance worsens volume stabilization according to Fig 4?*

We studied a possible effect of Gardos channels on human erythrocyte volume stabilization in the paper published in the Biophysical Chemistry, **80**, 199-215, 1999 [2]. Of course, opening of Gardos channels along can worsen the stabilization of human erythrocytes cell volume. And it happens probably in case of pathology named hereditary xerocytosis [3]. However, it was shown that in case of increase in non-selective cell membrane permeability the functioning of the Gardos channels can significantly improve the erythrocyte cell volume stabilization [2] (see lines 380-384 in Discussion).

References

1. Vitvitsky VM, Frolova E V, Martinov M V, Komarova S V, Ataullakhanov FI. Anion permeability and erythrocyte swelling. Bioelectrochemistry 2000;52:169–77.
2. Martinov M V., Vitvitsky VM, Ataullakhanov FI. Volume stabilization in human erythrocytes: Combined effects of Ca2+-dependent potassium channels and adenylate metabolism. Biophys Chem 1999; 80:199–215.
3. Gallagher P.G. Disorders of erythrocyte hydration. *Blood* 130, 2699-2708, 2017.

Reviewer #2

The paper by Ataullakhanov et al. describes the important role of the Na-pump as well as the transmembrane sodium and potassium gradients for volume stabilization at increased cation permeability. The obtained results are based on mathematical modelling. In addition to former investigations, the authors took into consideration glycolytic metabolites and adenine nucleotides. The paper is well written and the results are of importance for fundamental red blood cell research.

However, the paper would improve by taking into consideration the following points:

General:

*The authors state that the red blood cells change their shape when passing through narrow capillaries. Most mammalian red blood cells can transform from discocytes to echinocytes or stomatocytes. However, the red cells from camels are ellipsoid and do not change the shape. This should be added at least in parenthesis.*

Thank you for this correction. We mentioned this information in the Introduction (lines 57-67) with corresponding references.

*In the text (Results) a permeability increase of 5 times is mentioned, whereas in Fig. 2 a 4-fold increase is given in the legend.*

We note in the text (lines 172-175) that at 5-fold increase in cell membrane permeability erythrocyte gets a spherical shape and any further increase in the permeability causes the cell damage. In the Fig. 2 we demonstrate a kinetics of cell volume and intracellular [Na^+^] at cell membrane permeability increase (4-fold) that does not cause the cell destruction yet. In the revised version of the manuscript, we indicated limits of erythrocyte volume increase in Fig 2A and in new figures (Fig 6, and Fig 7).

*It is mentioned that a sodium and potassium gradient is existing in most mammalian cells. Investigating red blood cells, it should be mentioned that in most cases this is based on the sodium pump. However, dog and cat red cells do not have a sodium pump but have also such gradients. It is due to the existence of a Ca/Na exchanger (absent in other mammalian red blood cells), which produces based on the calcium pump a Na gradient and based on this Na gradient a Na, K,2Cl cotransporter produces a K gradient.*

We know about an “unusual” mechanism of the maintenance of transmembrane sodium ion gradient in dog and cat erythrocytes. We think that it would be very interesting to investigate how effective this mechanism is in maintaining a constant surface area to volume ratio. But this mechanism is not well studied to our knowledge. Also, it was shown recently, that at least in some dog breeds animals with normal transport Na/K-ATPase in erythrocytes exist, that maintain Na^+^ and K^+^ transmembrane gradients similar to other species [1-3]. This topic is beyond of aims of our study and we would not like to discuss it here.

*I would suggest to take out the statement given in lines 295 – 299. It would need more basic research on membrane ion transport processes.*

To our knowledge, there are no good rational hypotheses regarding necessity of two opposite transmembrane ion gradients with high intracellular concentration of K^+^ ions in majority of cells. We do not pretend to present a complete explanation of that phenomenon but, in our opinion, we offer a reasonable assumption about it and would like to mention it in the text.

*Furthermore, the authors take into consideration the Gardos channel as an additional ion transport system. However, there are much more cation transport systems in the red blood cell membrane. I would like to have a paragraph mentioning these systems and discussing a possible role in the effect investigated in the present paper. At least the authors should say that it is planned to take such ion transport systems into account.*

Thank you for this comment. We mentioned the cation transport systems presented in human erythrocytes in the Introduction (lines 120-122). Also, we mentioned in the Discussion section (line 384-386) the necessity to investigate a contribution of other ion transport system in erythrocyte volume stabilization.

Minor points:

*Throughout the paper “erythrocyte” or “red blood cells” should be used (Corrected). In addition, non-selective should be written with – throughout the paper (Corrected). Define A in mathematical equations* – A description of parameter A was added to the text (lines 435, 436).

*Line 28: the role* …- Corrected

*Line 105: Instead: … reduced significantly more. Better: …* *reduced to a greater extend*. - Corrected

*Line 122: … why an erythrocyte* …- Corrected

*Line 124: Delete space in Na/ K-ATPase* - Corrected

*Line 127: … of an erythrocyte*. - Corrected

*Line 131: Instead of disturbance –* *change of the permeability* - Corrected

Line 132: Instead of undergoes – underlie significant mechanical stress, ….-Corrected

*Lines 156, 348, 355 – comma before respectively*.- Corrected

*Lines 202, 234, 240 - Figs*. - Corrected

*Line 287: Instead of … damage to the cell membrane of these cells - … effect on the membrane of these cells* ….- We corrected the phrase in a different way (Line 350).

*Line 335: Comma before which* - Corrected

*Line 357 (and further on, e.g. 438): There is no °K – only K*. - Corrected

Line 418: 5.5 - Corrected

*Line 429: ATP not Russian ATF (actually, it was line 492)* - Corrected

*Table: 3 times parameter in Russian*. - Corrected

*References: Instead of brackets it should be stated (in Russian) – ref 3, 26, 33, 38.*- the brackets were added by the reference manager software (Mendeley). We stated that the referred papers are in Russian.

*What means n.d. in ref. 3?*

*Some references (titles) are written with capital letters (20, 32, 34, 36, 39). Most references, however with small letters.* – All issues regarding the References were corrected.

References

1. Maede Y., Inaba M., Taniguchi N. Increase of Na-K-ATPase activity, glutamate, and aspartate uptake in dog erythrocytes associated with hereditary high accumulation of GSH, glutamate, glutamine, and aspartate. *Blood* 61, 493-499, 1983.
2. Fujise H., Higa K., Nakayama T., Wada K., Ochiai H., Tanabe Y. Incidence of dog possessing red blood cell with high K in Japan and East Asia. *J. Vet. Med. Sci.* 59, 497-497, 1997.
3. Conrado F.O., Oliveira S.T., Lacerda L.A., Silva M.O.D., Hlavac N., Gonzalez F.H.D. Clinicopathologic and electrocardiographic features of Akita dogs with high and low erythrocyte potassium phenotypes. *Vet. Clin. Pathol.* 43, 50–54, 2014.

Sincerely yours

Victor M. Vitvitsky, D.Sc.

Center for Theoretical Problems of Physico-Chemical Pharmacology

Russian Academy of Sciences

Moscow 109029, Russia

E-mail: [victor_vitvitsky@yahoo.com](mailto:victor_vitvitsky@yahoo.com)
